# Robotic versus laparoscopic cholecystectomy for difficult gallbladders: an observational study of tertiary centre cases

**DOI:** 10.1007/s00464-025-11586-8

**Published:** 2025-03-20

**Authors:** Michal Kawka, Zaynab A. R. Jawad, David Hakim, Madhava Pai, Scarlet Nazarian, Tamara M. H. Gall, Christopher Wadsworth, David Nicol, Long R. Jiao

**Affiliations:** 1 St George’s Hospital, St George’s University Hospitals NHS Foundation Trust, London, UK; 2 Ealing Hospital, London North West University Healthcare NHS Trust, London, UK; 3 The Hammersmith Hospital, Imperial College Healthcare Trust, London, UK; 4 Department of Academic Surgery, The Royal Marsden Hospital, London, UK

**Keywords:** Robotic cholecystectomy, Laparoscopic cholecystectomy, Gallstones, Difficult gallbladder, Minimally invasive surgery

## Abstract

**Background:**

Although laparoscopic cholecystectomy (LC) is considered a low-risk procedure, intraoperative bleeding, bile duct injury and bile leak occur frequently in the ‘difficult’ gallbladder. Robotic cholecystectomy (RC) can overcome difficulties related to poor vision and instrumentation in difficult cases to avoid intraoperative complications and conversion to open surgery. The aim of the study was to evaluate the outcomes of laparoscopic and robotic cholecystectomy in patients with difficult gallbladders referred to a tertiary HPB centre.

**Methods:**

We conducted a retrospective review of all patients referred to a senior hepatobiliary and pancreatic surgeon with a ‘difficult’ gallbladder between December 2013 and March 2024. Primary outcomes were conversion to open procedure, and 30-day post-operative complications.

**Results:**

A total of 88 difficult gallbladder cases (*n* = 35 laparoscopic, *n* = 53 robotic) were referred to a tertiary HPB centre during the study period, consisting of 21.7% of cholecystectomies (*n* = 404). The total complication rate (14.3% vs 3.8%, OR 4.25, 95% CI 0.77–23.28, *p* = 0.0951) and conversion rate (8.6% vs 0.0%, OR 11.52, 95% CI 0.57–230.32, *p* = 0.109) were both higher in the laparoscopic group, but these differences were not statistically significant. The median operative time was significantly higher in the laparoscopic group (108.5 min vs 50.0 min, *p* = 0.001).

**Conclusions:**

Both robotic and laparoscopic cholecystectomy are viable approaches in difficult gallbladder cases, with robotic cholecystectomy being associated with potentially fewer complications and conversions to open surgery. Pre-operative referral of patients with difficult gallbladders and the intra-operative abandonment of difficult cases can both be considered safe exit strategies for difficult gallbladder cases.

Since the first laparoscopic cholecystectomy (LC) was performed in 1985, it has become the standard technique for elective cholecystectomies [1, 2]. While it is usually a low-risk procedure that is done on an ambulatory basis in many centres, complications such as bile duct injury or bile leak continue to occur in cases more commonly considered to be ‘difficult’ cholecystectomies [3, 4]. The incidence of the difficult gallbladder is reported to be as high as 16% [5, 6]. A small but significant proportion of these patients will develop long-term problems as a result of bile duct injury; these long-term problems include recurrent cholangitis and biliary stenosis [7]. Several studies have attempted to identify the pre-operative risk factors to predict difficult cases using the rate of conversion to open surgery as an indicator of difficulty. The factors identified include male gender, previous abdominal surgery, obesity and a contracted or thick walled gallbladder [8–10]. Scoring methods to predict the difficult laparoscopic cholecystectomy have also been reported [10, 11]. However, 30 years later, it remains challenging to determine with certainty which cholecystectomy cases will be deemed "difficult" until evaluation in the operating room.

Strategies to overcome the difficult gallbladder have been reported including identifying the ‘critical view of safety’ or performing a subtotal cholecystectomy [12, 13]. Despite this, in the UK controversy continues over whether laparoscopic cholecystectomy procedures should be performed only by HPB surgeons, or whether this surgical procedure can be performed with comparable outcomes by all general surgeons. In one study the authors reported lower rate of bile duct injury following cases performed by upper GI surgeons and HPB surgeons, compared with those by other surgeons [14]. What is more, with the emergence of robotic platforms, robotic cholecystectomy (RC) has also been suggested as a solution to minimise bile duct injury, and conversion rates in difficult cholecystectomies. This could be due to the inherent benefits a robotic platform offers with regards to clarity of view, due to 3D camera vision, and precision of dissection due to articulated endo-wristed instruments, both of which, could improve outcomes [15].

Our tertiary referral centre in West London has extensive experience with both LC and RC, using them to tackle difficult cholecystectomies. The aim is of this study was to describe the experiences managing difficult cholecystectomies, and to compare the outcomes of laparoscopic and robotic cholecystectomies.

## Methods

From December 2013 to March 2024, all patients aged over 18 years, referred to our tertiary HPB centre in West London, United Kingdom from other hospitals with anticipated, or identified difficult cholecystectomies were included. Difficult cholecystectomy cases were performed laparoscopically until the end of 2016, when the lead surgeon transitioned to performing robotic cholecystectomies, due to access to a surgical Da Vinci robot. As such, all patients who were accepted under the abovementioned criteria from January 2017 to March 2024 underwent robotic cholecystectomy. Patients with missing data were excluded from the outcome analysis. Difficult cholecystectomy was defined as: cases referred by another general surgeon following an attempted and abandoned cholecystectomy, subtotal cholecystectomy, or radiologically confirmed Mirizzi’s syndrome types II to V prior to cholecystectomy.

### Data collection

A retrospective review of the case notes and computerised patient was carried out. The following data were extracted from the medical records for each patient: age, sex, clinical presentation, initial surgical procedure, the reason for referral, prior surgery or radiological interventions, potential reasons for the abandonment of surgery, final histological diagnoses, and the duration of surgery. The following co-primary outcomes were collected: 30-day post-operative complication rate, and rate of conversion to open procedure. Reporting was in accordance with the STROBE statement for cohort studies [16]. The study was conducted in accord with the ethical standards of the Helsinki Declaration of 1975.

### Surgical technique

For robotic cholecystectomy (RC), patients were positioned in the supine position with 15 degrees reverse Trendelenburg, with pneumoperitoneum induced via a sub umbilical Hassan technique (Kii Balloon Blunt Tip system 12 × 100 mm, Applied Medical, Netherlands). The Si or X system were docked from the head of the patient, whilst the Xi system was docked from the patient’s right side. Similarly, for laparoscopic cholecystectomy (LC) we used a standard technique.

### Data analysis

The data were analysed using R version 4.3.0 (The R Foundation, Vienna). The LC and RC groups were compared with regards to outcomes and baseline characteristics; depending on distribution of data, continuous variables were compared using Mann–Whitney U, Kruskal Wallis, or Student’s *T*-Test, whilst categorical variables were compared using Chi-Square Test or Kruskal Wallis Test. *p*-value threshold for significance was set at 0.05.

## Results

### Baseline characteristics

During the first study period (2013–2016), a total of 177 patients were referred to our institution for laparoscopic cholecystectomy. Out of those, 35 patients (19.7%) were classified as having a ‘difficult’ gallbladder and were included in the analysis. During the second study period, (2017–2024), a total of 227 referred patients underwent robotic cholecystectomy. Out of those 53 patients (22.3%) were classified as having undergone a difficult gallbladder, and were included in the analysis. The baseline characteristics of the two subgroups are summarised in Table 1. The two cohorts were comparable in terms of sex, BMI, and pre-referral surgical and endoscopic management. However, patients who underwent RC were on average 12 years older than those in LC group. The robotic group had significantly more pre-referral radiological drainage procedures performed (28.3% vs 0.0%, *p* = 0.0002). This reflected the local policy used during the COVID-19 pandemic period when the prioritisation of surgery was given to cancer patients and non-cancer, benign gallbladder disease were manged non-operatively.

**Table 1:** Baseline characteristics of patients in laparoscopic cholecystectomy and robotic cholecystectomy groups

	LC (*n* = 35)	RC (*n* = 53)
Sex [male (%)]	8 (22.9)	15 (28.3)
Age [median (range)]	50 (25–81)	62 (35–89)
BMI [mean ± SD]	31.7 ± 5.5	33.0 ± 4.3
Pre-referral surgical management [*n* (%)]	10 (28.6)	16 (30.2)
Pre-referral radiological drainage [*n* (%)]	0 (0.0)	15 (28.3) **
Pre-referral endoscopic intervention [*n* (%)]	0.(0.0)	1 (1.8)

^*^ *p* < 0.05, ** *p* < 0.01, *** *p* < *0.001. LC* Laparoscopic cholecystectomy, *RC* Robotic cholecystectomy

In the LC group, 10 (28.6%) patients had undergone surgical management before referral; 6 patients (17.1%) had undergone an abandoned cholecystectomy, 2 patients had undergone a subtotal cholecystectomy, and 2 had a history of previous laparoscopic cholecystectomy with retained CBD stones, and presented with recurrent acute cholangitis at 3 and 5 months post-operatively. In the RC group, 16 patients (30.2%) had undergone an abandoned laparoscopic cholecystectomy. Moreover, in the RC group, 15 patients had image-guided drainage of the gallbladder before referral (28.3%), with one additional patient having had an ERCP guided stent inserted (1.8%).

### Reasons for referral

Reasons for referral are summarised in Table 2. In the LC group, complicated gallstone pancreatitis (*n* = 11, 31.4%, *p* = 0.003) was the leading reason for referral, compared to RC group, where Mirizzi’s syndrome was the leading reason (*n* = 15, 28.3%, *p* < 0.05). Once again, it reflects the change of practice in recent years by not taking on complex gallbladder cases for LC.

**Table 2:** Reasons for referral to tertiary centre, and timing of referral

Time of referral	LC (*n* = 35, %)	RC (*n* = 53, %)
Pre-operative	25 (71.4)	37 (69.8)
After surgical procedure	10 (28.6)	16 (30.2)
Reason for tertiary referral		
History of complicated gallstone pancreatitis	11 (31.4)	2 (3.7) ***
Repeated biliary colic	4 (11.4)	6 (11.3)
Acute cholecystitis	5 (14.3)	13 (24.5)
Mirizzi’s syndrome	6 (17.1)	15 (28.3)*
Perforated gallbladder/empyema	2 (5.7)	10 (18.8)
Suspected cholecystoduodenal fistula	2 (5.7)	2 (3.7)
Hepatic artery aneurysm	1 (2.9)	0 (0.0)
Suspected gallbladder cancer	4 (11.4)	5 (9.4)
Reason for pre-referral abandonment		
Extensive adhesions	8 (80.0)	12 (75.0)
Suspected cholecystoduodenal fistula	1 (10.0)	2 (12.5)
Suspected gallbladder malignancy	1 (10.0)	2 (12.5)

^*^ *p* < 0.05, ** *p* < 0.01, *** *p* < *0.001. LC* Laparoscopic cholecystectomy, *RC* Robotic cholecystectomy

### Short-term outcomes

Outcome comparisons are summarised in Table 3. The total complication rate was higher in the LC group (14.3% vs 3.8%, *p* = 0.074), however, this was not statistically significant. There were no bile leaks, or retained CBD stones in the RC group. There was no conversion to open cholecystectomy in the RC group, compared to 3 (8.6%) conversions to open surgery in the LC group. There was no mortality within 30 days of the procedure in either group. The median operative time was significantly longer for LCs (median 108.5 min vs 50.0 min, *p* = 0.001). The median length of stay was longer in the LC group compared to the RC group (median 2 days vs 1 day, *p* = 0.042).

**Table 3:** Short-term and operative outcomes

	LC (*n* = 35, %)	RC (*n* = 53, %)	OR (95% CI, *p*-value)
Total Complications	5 (14.3)	2 (3.8)	4.25 (0.77–23.28, *p* = 0.0951)
Bile Leak	1 (2.9)	0 (0.0)	4.65 (0.18–117.50, *p* = 0.350)
CBD stone	2 (5.7)	0 (0.0)	7.98 (0.37–171.49, *p* = 0.184)
Intra-abdominal collection	1 (2.9)	1 (1.9)	1.52 (0.09–25.28, *p* = 0.766)
Pancreatitis	1 (2.9)	0 (0.0)	4.65 (0.18–117.50, *p* = 0.350)
Wound infection	0 (0.0)	1 (1.9)	0.49 (0.01–12.44, *p* = 0.667)
Conversion to open	3 (8.6)	0 (0.0)	11.52 (0.57–230.32, *p* = 0.109)
Total Operative Time [median (range)]	108 (55–204)	50 (32–163)***	N/A
30-day mortality	0 (0.0)	0 (0.0)	N/A
Total length of stay (days) [median (range)]	2 (1–38)	1 (1–3)*	N/A

^*^= *p* < 0.05, ** = *p* < 0.01, *** = *p* < *0.001. LC* Laparoscopic cholecystectomy, *RC* Robotic cholecystectomy, *OR* Odds ratio

### Histology

The final histological diagnoses are summarised in Table 4. The majority of the LC group specimens showed chronic cholecystitis (68.6%), followed by acute on chronic cholecystitis (20.0%). This was mirrored in the RC group (chronic cholecystitis − 49.1% and acute-on-chronic cholecystitis − 45.3%, respectively). There were two adenocarcinomas of the gallbladder identified (5.7%) in the LC group and none in the RC group; this is despite there being cancers suspected within both patient groups.

**Table 4:** Final histological diagnosis

Histology	LC [*n* (%)]	RC [*n* (%)]
Chronic cholecystitis	24 (68.6)	26 (49.1)
Acute-on-chronic cholecystitis	7 (20.0)	24 (45.3)*
Gallbladder adenocarcinoma	2 (5.7)	0 (0.0)
Gangrenous gallbladder	2 (5.7)	3 (5.7)

^*^ = *p* < 0.05, ** = *p* < 0.01, *** = *p* < *0.001*. *LC* Laparoscopic cholecystectomy, *RC* Robotic cholecystectomy

## Discussion

A total of 404 patients underwent cholecystectomy at our center over the study period; 88 of these patients were referred to our tertiary referral HPB service because they were considered to be patient with "difficult" gallbladders. Of these difficult operations, 35 were performed laparoscopically, 19.7% of all laparoscopic cases (*n* = 177), and 53 were performed robotically, 22.3% of all robotic cholecystectomies (*n* = 227). The complication rate was higher in the LC group (14.3% vs 3.8%, OR 4.25, 95% CI 0.77–23.28, *p* = 0.0951), but the difference was not statistically significant. There were no bile duct injuries in either of the groups. A lower conversion to open surgery rate, and shorter length of stay were observed in the RC group.

In this series of difficult cases, there were no bile duct injuries in either LC and RC groups, compared to a rate of between 0 and 6% quoted in the literature [17]. This might be due to operative strategies of avoiding dissection in Calot’s triangle where this was felt to be unsafe, due to obliteration of normal anatomy secondary to previous surgery or inflammation. This was achieved by performing a combination of fundus-first dissection of the gallbladder and lateral approach to the gallbladder to free D2 and hepatic flexure of colon from the gallbladder and portal triads. However, a recent publication form the USA (1,001,004 LC vs 25,084 RC) showed an increased incidence of bile duct injury in the robotic group [18]. In that study patients having robotic assisted surgery had a higher comorbidity burden and a higher BMI compared with the laparoscopic group. As such, these patients might otherwise not have been a candidate for LC, or would have undergone open cholecystectomy. Furthermore, there are many reasons for biliary injury in patients with gallstone disease, the most common of which remains poor visualisation of the gallbladder and its surrounding vital structures during laparoscopy due to its inherent limitation relying solely on assistants to provide help with camera and retraction [5].

The rate of conversion in the LC group was 8.6% which is more in line with those patients undergoing simple, rather than difficult cholecystectomies. The rate of conversion for all cholecystectomies carried out in England over a 2-year period was reported as 5.6% whereas that for difficult cholecystectomies is reported to be as high as 30% [19]. The potential lower rates of conversion further supports the establishment of formal referral pathways to a tertiary HPB centres, as it translates to downstream reduction in hospital stay as well as benefiting the patient in terms of recovery time and earlier return to normal activities. Tertiary referral is therefore beneficial and crucial for difficult gallbladders identified on either preoperative imaging or, intraoperative laparoscopy during attempted cholecystectomy, to prevent serious complications.

The longer operative in LC group time goes against previously published literature on the topic. Although laparoscopic cholecystectomy has been previously reported to take less time when compared to surgical times for robotic surgery, this has often reflected the time associated with the RC learning curve or with the time required for robotic docking. Following the initial training period, we achieved a robotic docking times that were less than or equivalent to the time required for the laparoscopic setup. As such, the length of time reflected the true surgical time for these complex difficult cholecystectomies, showing that RC can be potentially done in shorter time than LC.

In this series intraoperative cholangiography (IOC) was not used. The proponents of IOC postulate it contributes to reduced risk of bile duct injury [20] through aiding in the intraoperative identification of common bile duct stones [21]. As bile duct injuries are rare, investigating the impact of IOC on bile duct injury has thus far been difficult to assess. Generally, it is a useful tool to delineate bile duct anatomy if used routinely, however, misinterpretation of the cholangiogram is a recognised pitfall even in the hands of experienced HPB surgeons, and its use during laparoscopic cholecystectomy is according to individual surgeons’ preference [22].

When comparing LC and RC, cost is an important consideration, which is particularly pertinent in the context of the UK, where the healthcare system has limited resources. Multiple studies report RC being associated with higher overall costs [23–25]. Yet, there exists marked heterogeneity in cost evaluation. What is more, with more robotic systems enteric the market worldwide and higher rates of adoption, economic advantage of LC could decrease over time, especially when consumables and set-up costs are lowered [26]. Questions still remain over costs of personnel training, and cost evaluations should be routinely included in studies evaluating new surgical techniques, such as RC, to ensure comprehensive assessment that can be applied across a range of healthcare systems.

Diagnostic laparoscopy as a tool in the stratification of the difficult gallbladder cases has not been previously described in the literature even though direct visualisation is the most reliable predictor of the difficult cholecystectomy, regardless of pre-operative, non-invasive imaging. In cases where LC was attempted but abandoned due to anticipated difficulties, most patients were discharged on the same day, and referred to our centre for further management. The most common reasons for abandonment were extensive adhesions to surrounding structures including the duodenum or the suspicion of malignancy. The data conflicts with previous studies that display an inclination to complete operative management for gallstones in one procedure [21, 22]. While this is feasible and safe in the majority of cases it should also be recognised that in some situations it is safest to abandon a procedure to allow the best course of treatment to be undertaken and that this should not be regarded as a failure of treatment. To identify the impact of this management protocol a larger series on only abandoned laparoscopic cholecystectomies needs to be conducted in the future. However, the current data supports further research into a formal referral pathways for difficult gallbladders identified pre-operatively, or intra-operatively, on initial laparoscopic assessment.

While this series raises some important points, one of the limitations of the study is that all the cases were referred to and carried out by one surgeon. As such, the rate of bile duct injury needs to be evaluated in a larger series of patients and units. What is more, for both laparoscopic, and robotic cholecystectomies, a learning curve effect could have influenced the outcomes. Although a RCT would be ideal to address those issues by providing level 1 evidence, our view is that it is unrealistic to conduct such a trial, and as such, a multicentre registry of difficult cholecystectomies would provide valuable real world evidence on current practices. Furthermore, almost all surgeons will switch from a laparoscopic technique to a robotic platform once proficiency in robotic skill is acquired. There have been few randomised trials on laparoscopic cholecystectomy versus open, and it is now the gold standard operation driven by surgical and technological innovation and patients’ demand for minimally invasive surgery. We also did not collect data on cost of LC and RC during the study period, which would have allowed for economic analysis.

Tertiary referral is a safe and feasible option for managing patients with difficult cholecystectomies. Further, larger registries need to be established to investigate contemporary practice, and lead towards a standardised referral criteria development. Whilst, both laparoscopic and robotic cholecystectomies are feasible strategies for difficult cholecystectomies, robotic platform is potentially associated with advantages such as shorter operative time, and a shorter length of postoperative stay.
